# Enhancing a Successful Pregnancy and Delivery After ICSI in Advanced-Age Woman with Concurrent Disorders: A Case Report

**Published:** 2020

**Authors:** Faranak Aghaz, Zahra Mokari, Mitra Bakhtiari

**Affiliations:** 1- Fertility and Infertility Research Center, Kermanshah University of Medical Sciences, Kermanshah, Iran; 2- Department of Social Sciences, Razi University, Kermanshah, Iran; 3- Infertility Research and Treatment Center, Motazedi Hospital, Kermanshah University of Medical Sciences, Kermanshah, Iran

**Keywords:** Advanced maternal age, Autologous oocytes, Embryo transfer, Intracytoplasmic sperm injection, Live birth

## Abstract

**Background:**

The objective of this case presentation was describing a live birth in an advanced-age woman with an extremely enlarged uterus, an ovary with blocked fallopian tubes, hypothyroidism and generalized anxiety disorder caused by child-birth following intracytoplasmic sperm injection/embryo transfer (ICSI-ET) with autologous oocytes.

**Case Presentation:**

A 47-year-old patient with an enlarged uterus due to recurrent multiple fibroids following myomectomy was referred to clinical laboratory with a high level of desire to follow the prescribed recommendations and approaches to retrieve her fertility. The patient underwent two cycles of oocyte retrieval and two rounds of frozen-thawed embryo transfer. To achieve a successful pregnancy after oocyte retrieval (birth weight of 3300 *g* at 38 weeks of gestation), a frozen/thawed embryo in the second cycle of ET was transferred.

**Conclusion:**

Usage of efficient planning and management of ICSI treatments in patient with autologous oocytes and concurrent disorders, can be used as a new approach to cure the affected individuals.

## Introduction

While the increasing world population appeares as an upcoming tsunami, the increased age of people can affect fertility of aged women unexpectedly. Also, a recent study reported that less than 20% of live births occurred in mothers aged 35 years and above (1, 2). A similar trend of higher maternal age has also been observed in low birth weight (VLBW, birth weight <1500 *g*) in preterm infants (2). From this point of view, the advanced maternal age (AMA) in both developed and developing countries is known as one of the most prominent challenges for at least two-thirds of reproductive clinicians to manage women’s fertility. It is estimated that more than 12.3% of aged women (>40 years) attend IVF clinics to recover their reproductive potency (3).

According to scientific reports, several issues can effectively delay the age-related fertility, including progressive depletion of ovarian follicular reserve, lower quality of oocyte, uterine fibroids, and hypothyroidism (4). Various studies showed a direct correlation between women’s age and infertility rate. AMA can also increase the associated risks with pregnancy. However, the women are not entirely aware of all age-related risks of pregnancy; thus their potential to have successful pregnancy outcomes may be decreased over time (5). Previous findings proved that assisted reproductive technology is an acceptable approach to be used for women with limited oocytes reserve. For example, Ajayi et al. (6) reported an incidence of a live birth following ICSI administration in oligospermia and azoospermia cases of Nigeria. However, Rani et al. (7) reported a rare case of live birth in a 50-year-old woman following in vitro fertilization-embryo transfer with autologous oocytes. Moreover, Orazulike et al. (8) reported a live birth case after IVF in a 53-year-old woman. But in the literature, the current study is the first case report of live birth in a 47-year-old woman after ICSI-ET with her oocytes along with concurrent disorders.

Among the factors that reduce women’s fertility rate, the uterine fibroids (Known as leiomyomas or myomas) are the important agents involved in women‘s infertility. Mainly, the abnormal growth of uterine smooth muscles causes noncancerous heterogeneous tumors, which generally appear in women with age >50 years during childbearing period (9, 10). The fibroids have a significant variation in size (From undetectable tissues with the naked eye to vast masses of tumorous colonies), location, and structure (From single to multiple). Although the women have uterine fibroids in their life, these dangerous tumors often represent no recognizable symptoms. Thus, determination of prevalence rate is considered a complex criterion for both physicians and patients. However, studies indicated that the increased numbers of submucosal and intramural fibroids are the main factors of early pregnancy loss and infertility in aged women (10, 11).

Another critical factor enhancing the quality of fertility is the normal function of thyroid glands (TGs) (12). Based on the published studies, the women with underactive TGs (Or hypothyroidism) are susceptible to the incidence of infertility and recurrent abortion (13). The most obvious complications in women with hypothyroidism are luteal phase defect, anovulatory cycle, absence of ovulation, inconsistency of sexual hormones and hyperprolactinemia (14). The oral treatment with hypothyroidism drugs for a period of 3 months to 1 year could show a high level of benefits to trigger the pregnancy in asymptomatic infertile women. In this case report, a 47-year old woman treated by ICSI-ET by autologous oocytes who had a live healthy birth (38 weeks and baby weighing of 3.3 *kg*) by cesarean section was discussed. This case is spectacularly unique because the pregnant mother suffered from concurrent disorders.

The patient had hypothroidism for the past 15 years, which was controlled by administration of levothyroxine as T3 and T4 stimulating hormone.

## Case Presentation

A 47-year-old woman with a 7-year history of secondary infertility was referred to a clinical laboratory (Motazedi Infertility Center of Kermanshah) in May 2016. She got married at the age of 24 years with history of two pregnancies at the ages of 24 and 28. After the second birth, tubectomy was performed in order to prevent further pregnancies, and the right ovariectomy was also applied due to the right ovarian cysts. After the death of the first child, tuboplasty procedure was performed to reopen the fallopian tubes, but according to the hysterosalpingography results, the left fallopian tube was tortuous and blocked. At the time of referral to the medical center for improvement of fertility, she only had a blocked ovary.

**Figure 1.**
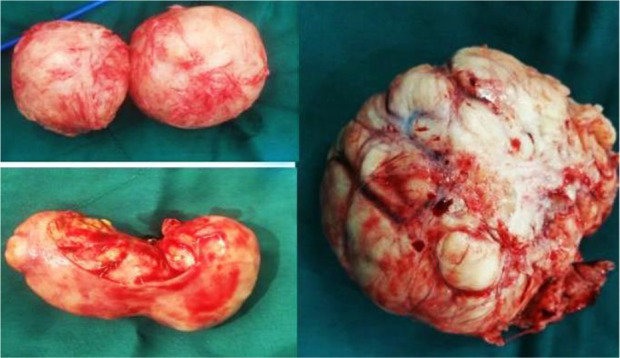
Different myomas; A: 3 *cm*, B: 8 *cm*, D: 18 *cm*

After tuboplasty, two natural pregnancies happened in which both of them ended with miscarriage. Six months later, the transvaginal ultrasound results indicated a further enlarged uterus (106×97×94 *mm*^3^) with more than three intramural uterine fibroids with maximum 20 *mm* diameters in size (Figure 1). In February 2010, by the use of abdominal hysteromyomectomy, all available fibroids were removed. Three months later, the patient attended to clinical laboratory with high interest to become pregnant. The menstrual cycles were regular, while the medication for hypothyroidism had been conducted over the past 15 years. The TSH level was controlled with the administration of levothyroxine (Using 1.74 *ng/day* dose). Semen analysis of her husband approved the status of normozoospermia with moderate oligospermia.

According to the patient history and also the results obtained from relevant laboratory examinations like antimüllerian hormone (AMH) as an indicator of ovarian reserve (AMH was 1.2 *μg/L* in this case), the patient was considered as a candidate for oocyre donation. During the first IVF cycle in November 2012, the patient underwent ovarian stimulation with human menopausal gonadotrophin (Gonal, HMG, Menogon), 450 *IU* daily from second to the twelfth day of menstrual cycle. The daily HMG concentration was increased to 750 *IU* for three additional days. A transvaginal ultrasound was performed on twelfth day and endometrium thickness was detected 11 mm. When two or more follicles reached a maximum diameter of 18 *mm*, 250 *μg* of human chorionic gonadotrophin (HCG) (Profasi HP; Serono), 5000 *IU* was administered. Then, transvaginal oocyte retrieval was performed 32–34 *hr* following hCG injection. After 36 *hr*, five oocytes were retrieved, and three of them were found in metaphase II step. These inefficient oocytes were granulated, which had no potential to produce an embryo. After two menstrual periods, the second treatment was applied using letrozole (Balkan Pharmaceuticals, USA) and then followed by stimulation with recombinant FSH (Gonal F) and HMG (Menopur) for 150 *mg/day* from second day to sixth day of the cycle. On the fourteenth day, the patient possessed several follicles, including an 18 *mm*-follicle, another with 14 *mm* and three others of 10 *mm*, in which four follicles were punctured, but no oocytes were retrieved.

In December 2013, after two menstrual periods, the IVF was performed for the third time by human menopausal gonadotrophin (Gonal, Menopur, Menogon), 150 *mg/day* from day 2 to day 6 of the cycle. On the fourteenth day, the patient possessed one follicle of 18 *mm* in diameter, another with 14 *mm* and three of them of 10 *mm*, and a trilaminar endometrial stripe of 9 *mm*. In the next step, the final maturation was enhanced with HCG (Profasi HP; Serono), 4500 *IU*. After 36 *hr*, one of the oocytes was transvaginally retrieved under ultrasound guidance. It was inseminated by conventional ICSI in which a two-cell embryo was obtained (Grade A).

In February 2014, the patient demanded to receive the first cycle of frozen-thawed-ET. For this purpose, to reduce the endometrial thickness, E2 (2 *mg* daily) was administrated for 3 months. The endometrial thickness was detected 8 *mm* by the use of transvaginal ultrasound which was inappropriate for pregnancy. The frozen-thawed-ET was canceled due to the lack of suitable conditions to maintain the protocol. After two menstrual periods, the second treatment was done with Suprefact (0.2 *mg* daily from day 9). In the twelfth day of menstrual cycle, when the endometrial thickness was completely prepared, a blastocyst (Grade A) was implanted in uterine cavity using a transcervical route.

The luteal phase continued with intramuscular injection of natural progesterone in oil solution (Prontogest, AMSA, Rome, Italy) with following doses of 40, 60, 80, 80, 80, and 80 *mg/d* in the next 6 consecutive days, respectively. Fifteen days after embryo transfer, β-human chorionic gonadotropin (β-HCG) test was positive. Intrauterine single fetal heartbeat was confirmed by ultrasound 40 days after ET. However, the first-trimester screening was performed at week 11 for nuchal translucency (1.6 *mm*), and a double marker assay was found normal. To detect all anomalies, the ultrasonography was repeated in the second trimester at week 20 of gestation. Fetal growth and amniotic fluid volume were in normal range. A third-trimester scan at week 28 of gestation showed normal amniotic fluid index and fetal growth parameters. At the twenty-eighth week of pregnancy, the patient complained about runny nose, and spots and the rest of the period continued with complete rest and progesterone 50 *mg* IM every 12 *hr*. Finally, the pregnancy process was terminated by emergency cesarean section due to probable occurrence of eclampsia at 38 weeks of gestation. A healthy female baby weighing 3.3 *kg* was successfully delivered.

### Ethical Consideration:

The current study was completely approved by the local ethical committee of Kermanshah University of Medical Sciences. The datasets used for the current study are not publicly available due to regulatory conditions concerning data usage and storage.

Written and verbal consent for the publication of this case report along with any additional related information was taken from the patient of this study.

## Discussion

Although recent advances in medical procedures considerably increased the quality of human health, the prevalence of human diseases was also increased in many cases and complicated health problems have appeared. Among all complications and disorders in human society in the past decades, the pregnancy complications are the most commonly diagnosed issues. Advanced paternal age is associated with perinatal mortality, intrauterine fetal death, VLBW, preterm infants and neurodevelopmental disorders like pre-eclampsia (15). An extensive PubMed search was performed for women with age of >40 years and experience of live birth with autologous oocytes concluding that the percentage of these infertile women treated by ICSI is increasing, particularly in countries where the egg donation is a cultural, religious, or ethical limitation (15). Few number of previously published papers suggested that a chance for successful ICSI with autologous oocytes is observed up until the end of 45th year (16, 17). In some studies, the pregnancies achieved by IVF procedure (7, 8, 17, 18) were all in women aged ≥44 years while all these cases never had concurrent disorders in oocyte retrieval. However, the current study is the first report of a successful pregnancy and live birth along with various pathologic conditions including extremely enlarged uterus, recurrent multiple intramural fibroids, an ovary with blocked fallopian tubes, hypothyroidism and generalized anxiety disorder caused by childbirth. This procedure was applied based on ICSI treatment in a 47-year-old woman with her oocytes from ICSI-ET.

The patient had recurrent multiple intramural fibroids. It is generally accepted that the presence of submucosal or intramural fibroids (19) have adverse effects on implantation, pregnancy and live birth rates in the assisted reproduction cycles. These complications appear to be solved following surgical removal of fibroids. Furthermore, the current observations approved this fact that the intramural fibroids increase the rate of spontaneous abortion in IVF and ICSI treatments (20). The current patient underwent myomectomy at the age of 45 years and delivered a healthy baby when she was 47. This report approved that a successful pregnancy after IVF with autologous oocytes can be achieved in women ≥45 with intramural fibroids and an ovary with blocked fallopian tubes in which the results should be considered as an amazing and impressive phenomenon.

On the other hand, other important factors including hypothyroidism (12) and generalized anxiety complications (21) that existed in this patient also displayed the potential effect on outcomes of assisted reproductive treatment. Thyroid hormones significantly showed the effective results in reproduction and pregnancy. Hypothyroidism is related to the increased level of thyrotropin-releasing hormone (TRH) production which stimulates the pituitary to secrete T3 and T4 affecting reproduction and pregnancy (12). Thyroid dysfunction is a common cause of infertility; thus the physiological level of TSH is a pre-requisite for fertilization. Thus, to manage the infertility of this patient, the TSH level was controlled by the use of levothyroxine.

## Conclusion

Currently, the success rate of ICSI treatment in women aged ≥40 years is low, and the best management for these patients is application of oocyte donation protocols. In these infertile women who used autologous oocytes, the request for ICSI treatment is increasing, particularly in countries where the oocyte donation has cultural, religious, or ethical limitations. Thus, in cases similar to the present patient with autologous oocytes and concurrent disorders, the planning and effective treatment and/or management can be used for successful outcomes. The successful live birth reported in this study can provide the assurance to have healthy children either by giving hope or guiding researchers for further experiments for other similar cases.
